# The relationship between fasting plasma glucose and MPO in patients with acute coronary syndrome

**DOI:** 10.1186/s12872-015-0088-z

**Published:** 2015-08-25

**Authors:** Xiangyu Zhang, Lini Dong, Qiong Wang, Xiaomei Xie

**Affiliations:** Department of Geriatrics, Second Xiangya Hospital of Central South University, Changsha, Hunan 410011 P. R. China

## Abstract

**Background:**

Inflammation plays a critical role in the progression of atherosclerosis, and hyperglycemia is a common feature in patients with ACS. We investigated the relationship between fasting plasma glucose (FPG) levels and the levels of the inflammatory factor, myeloperoxidase (MPO), in patients with acute coronary syndrome (ACS).

**Method:**

A total of 85 patients with no prior history of diabetes mellitus were recruited. The patients were divided into three groups based on their FPG levels as follows: group A, FPG < 5.6 mmol/l; group B, 5.6 mmol/l ≤ FPG < 6.1 mmol/l; and group C, FPG ≥ 6.1 mmol/l. The FPG concentrations and plasma MPO levels were determined, coronary angioplasty was performed, and the Gensini scores were used to evaluate the severity of the coronary lesion. The MPO expression in peripheral blood mononuclear cells (PBMCs) in patients with ACS was determined using western blot analysis.

**Result:**

The results demonstrated that the levels of FPG were significantly and positively correlated with plasma MPO levels, Gensini scores, high sensitive C reaction protein(hs-CRP)levels, leukocyte and neutrophils count. In multivariate regression analyses the FPG levels were positively correlated with plasma MPO levels, Gensini score and hs-CRP. The plasma MPO levels in the group C [68.68(52.62–91.88) U/L] were significantly higher than in the group A [63.04(26.18–97.75) U/L] and group B [58.22(23.95–89.54) U/L]. The plasma hs-CRP concentrations are also higher in group C [42.28 (0.31–169.40) mg/L] than in the group A [12.51(0.28–176.25) mg/L] and group B [14.7 (0.14–89.68) mg/L].

**Conclusion:**

This study demonstrates that FPG values are positively correlated with plasma MPO levels, suggesting MPO may play a role in the proatherogenesis of high FPG.

## Background

Increased plasma glucose is a common occurrence during the first few hours of acute coronary syndrome (ACS), not only in diabetics, but also in non-diabetic patients [1]. Epidemiological studies indicated that hyperglycemia plays an independent role in cardiovascular disease [2]. Hyperglycemia, regardless of diabetic status, remains to be a risk factor for the short-term mortality of patients with acute myocardial infarction and treated with percutaneous coronary intervention [3–5], whereas fasting plasma glucose (FPG) levels are a better predictor of adverse outcomes in ACS patients during hospitalization than the admission plasma glucose (APG) level [6]. Several studies showed that the proatherogenic role is related to the production of reactive oxygen species [6–8] and platelet dysfunction [5]. However, the direct influence, if any, of hyperglycemia, on ACS patients remains unclear.

Myeloperoxidase (MPO) is a type of leukocyte enzyme that is promptly released after activation. MPO and its oxidant products have been identified in atherosclerotic plaques, promoting a number of pathological events that participate in plaque formation and rupture [9, 10]. In clinical studies, elevated MPO levels are associated with an adverse prognosis and the occurrence of major cardiovascular events [11–13]; therefore, MPO is also a key inflammatory factor in the course of the plaque formation and rupture, similar to CRP. To our knowledge, there is no available study about the relationship between the fasting plasma glucose level (FPG) and MPO in patients with ACS. The objective of this study is to determine whether the changes in FPG influence MPO.

## Methods

### Study subjects

A total of 85 patients with acute coronary syndrome, including acute myocardial infarction and unstable angina, and no prior history of diabetes mellitus were recruited. The patients were divided into three groups based on their FPG levels as follows: group A (*n* = 33), FPG < 5.6 mmol/l; group B (*n* = 23), 5.6 mmol/l ≤ FPG < 6.1 mmol/l; and group C (*n* = 29), FPG ≥ 6.1 mmol/l. Unstable angina was defined as ischemic chest pain at rest, accompanied by transient ST-T segment depression and/or T-wave inversion within the next 24 h. The diagnosis of acute myocardial infarction was based on a history of ischemic chest pain >30 min, characteristic ECG changes, and an increase of creatine kinase activities to at least twice the normal upper level within 24 h of the pain. The exclusion criteria includes body temperature >38 °C; inflammatory diseases, such as infections, malignancies, and autoimmune diseases; human immunodeficiency virus (HIV); impaired liver function; renal failure; serum total cholesterol concentration >7.0 mmol/L; hemoglobin A1c (HbA1c) above 6.5 %; and recent major surgery. Experienced interventional cardiologists performed all revascularization procedures. Coronary angioplasty was performed and stent was implanted if necessary and the Gensini scores were used to evaluate the severity of the coronary lesion. The Institutional Ethics Committee of the 2nd Xiangya Hospital of Central South University approved this study. All of the subjects have provided written informed consent.

### Biochemical analysis

Using standard automated enzymatic methods, a Hitachi 912 automated analyzer, and reagents from the Kamiya Biomedical Company, the serum triglycerides (TG), total cholesterol (TC), high-density lipoprotein cholesterol (HDL-C), high sensitive C reaction protein (hs-CRP) and fasting plasma glucose levels (FPG) (FPG was determined after >8 h of fasting) and liver and renal functions were determined at the central chemistry laboratory of our hospital. The LDL-C level was calculated using the Friedwald formula, and the plasma MPO levels were measured with an ELASA kit (Jingmei BioTech Co.Ltd).

### Peripheral blood mononuclear cells preparation

Peripheral blood mononuclear cells (PBMCs) isolation was performed using the Ficoll-Hypaque density-gradient centrifugation method from the patients with ACS. After centrifugation, buffy coats were collected and washed in phosphate-buffered saline (PBS; Gibco, Darmstadt, Germany) for 3 times and suspended at a concentration of 2 × 10^6^ cells per milliliter with complete RPMI 1640 medium containing 2 mM l-glutamine, 100 U/mL penicillin, 100 μg/mL streptomycin, 0.05 mM 2-mercaptoethanol, supplemented with 10 % fetal calf serum (Gibco, Germany). Cell viability was evaluated using 95 % by the trypan blue exclusion test. Cells were collected for MPO protein assays.

### Detection of MPO expression in PBMCs by western blot

To determine the expression of MPO in human PBMCs, immunoblot analysis was performed. Cells (2 × 10^7^) were lysed with Triton lysis buffer (50 mM Tris-HCL, pH 8.0 containing 150 mM NaCl, 1 % Ttriton X-100, 0.02 % soldium azide, 10 mM EDTA, 10 μg/ml aprotinin, and1μg/ml aminoethyl benzenesulfonyl fluoride). Protein concentrations were determined by Bradford assay. One hundred micrograms of protein from the cell lysates were loaded onto a 7.5 % SDS-PAGE gel. After electronphoresis, the SDS-PAGE separated proteins were transferred to a nitrocellulose membrane (Amersham Pharmacia Biotech). The membrane was blocked with 2.5 % nonfat milk in PBS and incubated with rabbit antibody against human MPO (1:200 dilution) in PBS for 2 h. Then the membrane was incubated with goat anti-rabbit IgG conjugated with horseradish peroxidase (Santa Cruz, Biotechnology, CA, USA) at 1:1000 in PBS for 1 h. Blots were processed using an ECL kit (Santa Cruz) and exposed to X-ray film.

### Statistical analyses

All data were tested for normal distribution with Kolmogorov-Smirnov test and for homogeneity of variances with Levene’s test. Data are expressed as mean ± SD or median for parametric and nonparametric data respectively, categorical variables as numbers and percentages. The baseline characteristics of group categorized by FPG level were compared by use of ANOVA for continuous variables and by Kruskal-Wallis Test followed by Mann-Whitney nonparametric test for noncontinuous variables. Univariate and multivariate analyses of the effects of the factors on FPG were performed with linear regression analyses. Differences were considered statistically significant at the 2-sided *P* < 0.05 level. Statistical analyses were performed with SPSS statistical software (18.0).

## Results

### Baseline characteristics of the patients

The clinical characteristics of ACS were shown in Table 1. Leukocyte count in the group B and C were more than in the group A. The Gensini scores in the group B and C were significantly higher than in the group A. There were no statistically significant differences between the three groups in the other patient’s characteristics, medication histories and smoking.

**Table 1:** Baseline characteristics of the patients

	Group A	Group B	Group C
	*N* = 33	*N* = 23	*N* = 29
Age (year)	63 ± 10	68 ± 9	67 ± 10
Male/female(n)	18/15	12/11	20/9
BMI(kg/m^2^)	23.6 ± 0.4	23.5 ± 0.52	23.6 ± 0.3
Smoking, n (%)	20(61)	13(56)	19(69)
SBP(mmHg)	130 ± 4	127 ± 3	128 ± 3
DBP(mmHg)	76 ± 2	74 ± 2	75 ± 2
Leukocyte ( 10^9^/L)	5.5 ± 1.95	6.7 ± 1.87^*^	7.18 ± 2.10^*^
Creatinine (μmol/L)	109.7 ± 9.6	99.38 ± 4.5	99.65 ± 5.88
TG(mmol/L)	2.05 ± 0.25	1.85 ± 0.22	2.8 ± 0.90
TC(mmol/L)	5.05 ± 0.18	4.54 ± 0.18	4.8 ± 0.12
LVEF (%)	0.59 ± .03	0.58 ± 0.02	0.57 ± 0.03
Gensini score	29.7(21.0–56.2)	35.1(23.8–55.0)^*^	43.6(22.0–56.0)^*, **^
Medications, n (%)			
Aspirin	13 (39)	10 (43)	11 (37)
β-blocker	15 (45)	11 (47)	14 (48)
ACEI/ARB	21 (67)	16 (76)	20 (68)
CCB	7 (22)	4 (19)	5 (17)
Nitrate	21 (63)	16 (69)	18 (62)
Statin	10 (30)	8 (34)	11 (37)

Values are presented as mean ± SD. or *n* (%) or median

*BMI* body mass index; *SBP* systolic blood pressure; *DBP* diastolic blood pressure; *TG* triglyceride; *TC* total cholesterol; *LVEF* left ventricular ejection fraction; *ACEI* angiotensin-converting enzyme inhibitors; *ARB* angiotensin II receptor blockers; *CCB* calcium channels blockers

*P < 0.05 when compared with Group A; ** *P* < 0.05 when compared with Group B

### Correlation analysis FPG with other parameters

Spearman correlation analyses were performed to assess the relationships at baseline in all patients. The results demonstrate that FPG were significantly and positively correlated with plasma MPO levels, Gensini scores, hs-CRP levels, leukocyte count and neutrophils count (Table 2). Multivariate regression analyses were further performed to assess the relationships between the FPG with these variables and showed that the FPG levels were still positively correlated with plasma MPO levels, Gensini score and hs-CRP (Table 3).

**Table 2:** Correlation between the FPG with other variables in the patients

Variables	r	p
MPO	0.492	0.000
Gensini score	0.568	0.000
Leukocyte count	0.353	0.001
Neutrophil count	0.220	0.048
hs-CRP	0.453	0.001
TG	−0.37	0.734
TC	0.014	0.900
Creatinine	0.021	0.852
LVEF	−0.228	0.146
SBP	−0.095	0.397
DBP	−0.07	0.950
Age	−0.158	0.160
Gender	−0.068	0.546

*MPO* myeloperoxidase; *hs-CRP* high sensitive C reaction protein; other abbreviations are as shown in Table 1

**Table 3:** Multiple stepwise regression analysis of the correlation of FPG with hs-CRP, MPO and Gensini score

Independent variable^a^	Partial regression coefficient	Standard error	Standardized regression coefficient	T	P
Constant	2.339	0.615		3.802	0.000
Hs-CRP	0.014	0.004	0.306	3.588	0.001
MPO	0.024	0.005	0.346	4.439	0.000
Gensini score	0.051	0.015	0.279	3.359	0.001

### Effects of FPG on plasma MPO levels and hs-CRP in patients

**Fig. 1:**
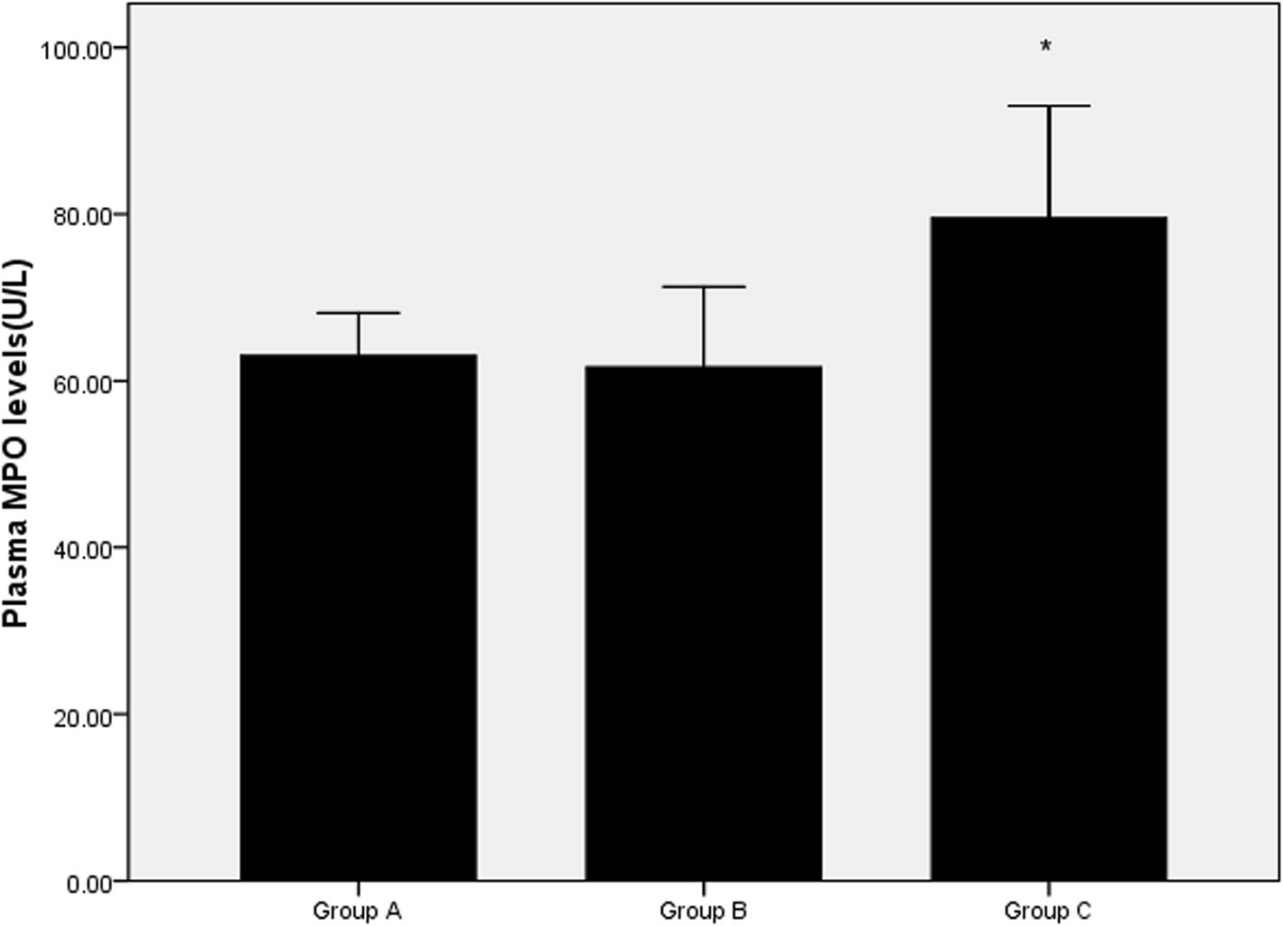
The plasma MPO levels in the three groups. ^*^ *p* < 0.05 when compared to Group A or Group B

**Fig. 2:**
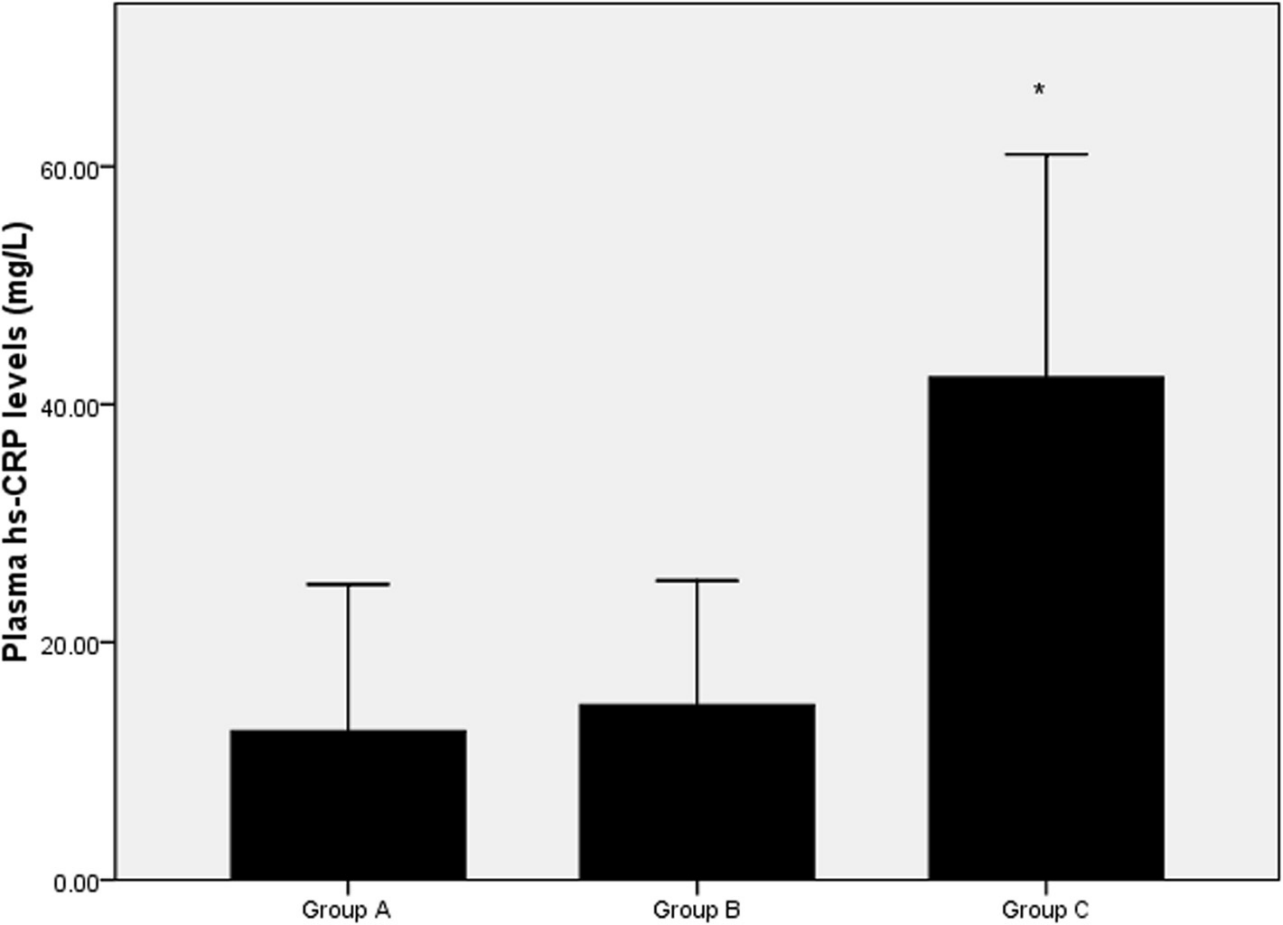
The plasma hs-CRP levels in the three groups. ^*^ *p* < 0.05 when compared to Group A or Group B

As shown in Figs. 1 and 2, the plasma MPO levels in the group C [68.68(52.62–91.88) U/L] were significantly higher than in the group A [63.04(26.18–97.75) U/L] and group B [58.22(23.95–89.54) U/L]. The plasma hs-CRP concentrations are also higher in group C [42.28 (0.31–169.40) mg/L] than in the group A [12.51(0.28–176.25) mg/L] and group B [14.7 (0.14–89.68) mg/L]. There were no significantly differences in these two parameters between in the group A and B.

**Fig. 3:**
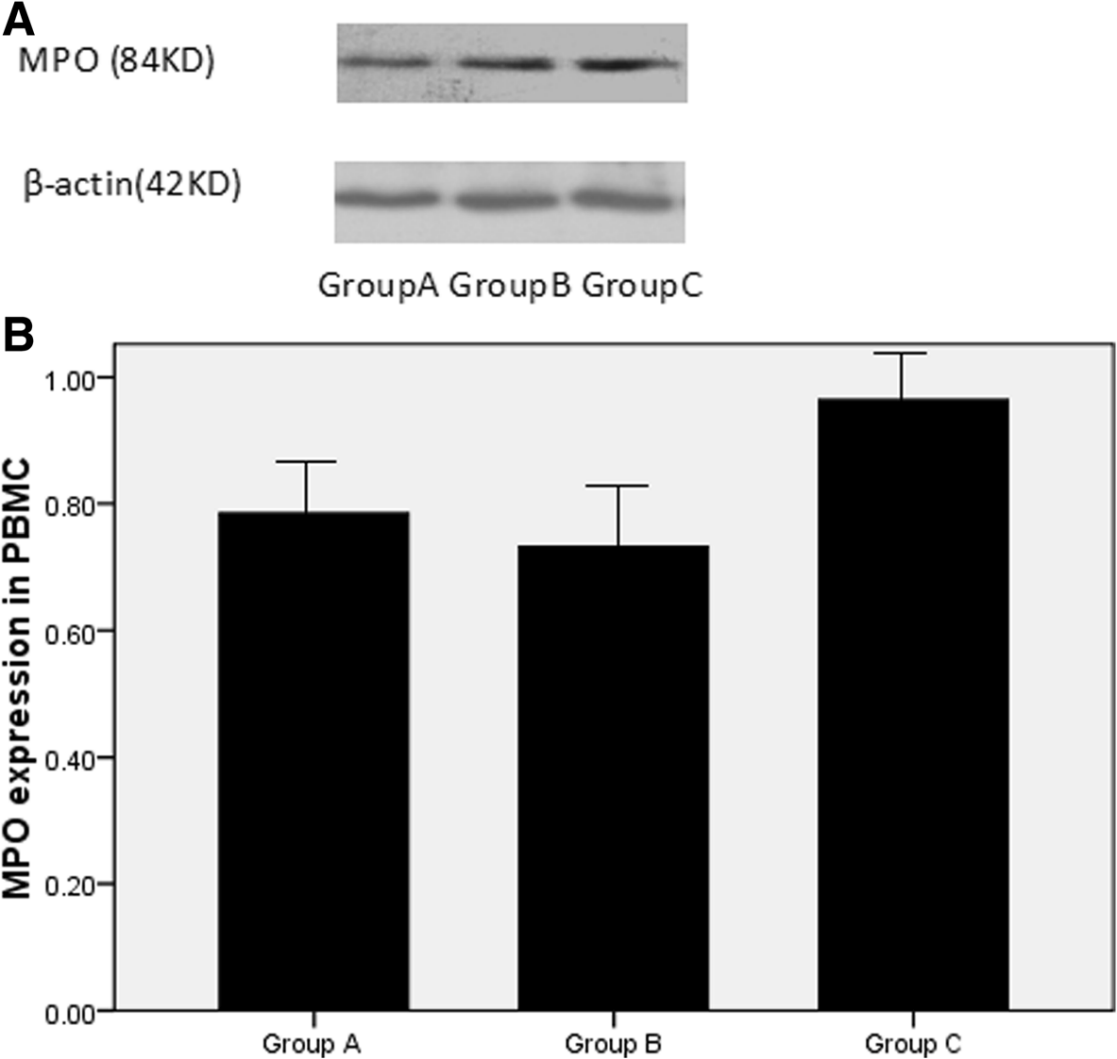
The MPO expression of PBMCs in the three groups. **a** Detection of MPO expression in cultured PBMCs from patients with ACS by Western blot analysis; **b** Effect of FPG on MPO expression of PBMCs, MPO expression is expressed as absorbance ratio of MPO product to β-actin product

### Effect of FPG on MPO expression of PBMCs in patients

MPO expression in PBMCs was detected by western blot analysis. As shown in Fig. 3, it was found that FPG had no effect on MPO expression in PBMCs from patients with ACS.

## Discussion

The blood glucose concentration is closely associated with cardiovascular diseases and a greater number of adverse events. The mechanism by which glycemia is associated with higher mortality remains unclear. Several studies showed that hyperglycemia is associated with increased coagulability and oxidative stress and promotes apoptosis in the rat heart [14–17]. The main finding of present study is that in patients with ACS, the FPG concentrations are positively correlated with plasma MPO levels. Furthermore, the plasma MPO levels and hs-CRP concentrations significantly increase in the highest FPG group (group C). These results demonstrate that high FPG may promote an inflammatory reaction during ACS, leading to an increase of plasma MPO levels, and finally causing plaque formation and rupture. As we all known, PBMCs are more important in atherosclerosis. However, the present study did not shown the significant effects of FPG on the MPO expression of PBMCs, indicating that FPG may play a role in the acute inflammatory reaction in patients with ACS via another type of leukocyte (maybe neutrophil). Being the most abundant leukocyte in the circulation, neutrophil received little attention in the pathophysiology of atherosclerosis, but recent advances pointed out a contributory role of neutrophils during atherogenesis and plaque destabilization [18–20]. Several proteins expressed and released by neutrophils emerged as possible biomarkers. MPO was abundantly expressed in neutrophil granules and partially released on neutrophils activation, leading to the plaque instability and vulnerability. In our study, the result also showed the positive association between the numbers of neutrophils and the FPG concentrations, this was consistent with the previous studies [19, 21–23].

In this study, we evaluated the relationship between the severity of coronary atherosclerosis and FPG, and the results showed that the FPG concentration increased with increasing Gensini score, suggesting that the FPG is also an indicator of the severity of the coronary artery lesion, which is consistent with the previous investigations [24, 25].

The majority of published studies evaluated the influence of APG on the prognosis of patients with ACS [26–28]. However, it is suggested that FPG may play a determining role. Otten et al. [29] showed that FPG is an independent predictor of adverse events in patients with ACS. In addition, Ravid et al. [30] and Soler et al. [31] demonstrated that FPG is a significant risk factor, and David Vivas et al. [6] have suggested that the FPG concentration is a better indicator of metabolic status than APG during the first few days of ACS. In addition, a significant relationship between high FPG and mortality within 30 days in non-diabetic patients with ACS was found by Suleiman et al. [32], with death usually occurring in patients with high FPG and normal APG values, or in those with high FPG and high APG, rather than in those with normal FPG. Moreover, for patients presenting an unfavorable clinical course during the first few hours of ACS, an increase in FPG may reflect a worse metabolic state and may be associated with more severe situations [33–35]; therefore, FPG may better represent the metabolic status of the patient with ACS. For clinicians caring for a patient with ACS, it is important to define the target below which the blood glucose levels should be maintained. The results of the study show that when the FPG levels are greater than 6.1 mmol/l (group C), the MPO levels and the hs-CRP levels are increased, indicating that clinicians may choose appropriate FPG values to avoid excessive inflammation in patients with ACS.

### Limitations

An important limitation of this study is that there was a small number patients, no patients were administered an OGTT. As a result, it cannot be determined whether patients with no prior history of diabetes will become diabetics during observation.

## Conclusions

This study demonstrates that FPG values are positively correlated with plasma MPO levels, suggesting MPO may play a role in the proatherogenesis of high FPG. The exact roles of high FPG, MPO and their relation in the atherosclerosis have been completely clarified, it needs further investigation.

## Abbreviations

FPG: Fasting plasma glucose
APG: Admission plasma glucose
MPO: Myeloperoxidase
ACS: Acute coronary syndrome
PBMCs: Peripheral blood mononuclear cells
hs-CRP: High sensitive C reaction protein
BMI: Body mass index
TG: Triglyceride
TC: Total cholesterol
SBP: Systolic blood pressure
DBP: Diastolic blood pressure
LVEF: Left ventricular ejection fraction
